# Extrafine Beclomethasone/formoterol compared to Fluticasone/salmeterol Combination Therapy in COPD

**DOI:** 10.1186/1471-2466-14-43

**Published:** 2014-03-12

**Authors:** Dave Singh, Gabriele Nicolini, Eddi Bindi, Massimo Corradi, Daniele Guastalla, Jorg Kampschulte, Władysław Pierzchała, Abdullah Sayiner, Mária Szilasi, Claudio Terzano, Jørgen Vestbo

**Affiliations:** 1 University of Manchester, Medicines Evaluation Unit, University Hospital of South Manchester Foundation Trust, Manchester, UK; 2 Corporate Clinical Development, Chiesi Farmaceutici S.p.A, Parma, Italy; 3 Department of Clinical and Experimental Medicine, University of Parma, Parma, Italy; 4 Corporate Marketing, Chiesi Farmaceutici S.p.A, Parma, Italy; 5 Praxis Dr. Jorg Kampschulte Wulffstrasse 7, Berlin, Germany; 6 Niepubliczny Zakład Opieki Zdrowotnej “PROFILAKTYKA”, ul. Medyków 10, 40-752 Katowice, Poland; 7 Department of Chest Diseases, Ege University Faculty of Medicine, Bornova, Izmir, Turkey; 8 Debreceni Egyetem Orvos és Egészségtudományi, Centrum-Tüdőgyógyászati Klinika Nagyerdei krt. 98, Debrecen 4032, Hungary; 9 U.O.C. Malattie Respiratorie Policlinico Umberto I- Università degli Studi di Roma “La Sapienza”, Roma, Italy; 10 Department of Respiratory Medicine, Odense University Hospital / University of Southern Denmark, Odense, Denmark

## Abstract

**Background:**

The study evaluated the efficacy of beclomethasone dipropionate/formoterol fumarate (BDP/FF) extrafine combination versus fluticasone propionate/salmeterol (FP/S) combination in COPD patients.

**Methods:**

The trial was a 12-week multicentre, randomised, double-blind, double dummy study; 419 patients with moderate/severe COPD were randomised to BDP/FF 200/12 μg or FP/S 500/50 μg twice daily. The primary objective was to demonstrate the equivalence between treatments in terms of Transition Dyspnoea Index (TDI) score and the superiority of BDP/FF in terms of change from pre-dose in the first 30 minutes in forced expiratory volume in the first second (FEV_1_). Secondary endpoints included lung function, symptom scores, symptom-free days and use of rescue medication, St. George’s Respiratory Questionnaire, six minute walking test and COPD exacerbations.

**Results:**

BDP/FF was equivalent to FP/S in terms of TDI score and superior in terms of FEV_1_ change from pre-dose (p < 0.001). There were no significant differences between treatments in secondary outcome measures, confirming overall comparability in terms of efficacy and tolerability. Moreover, a clinically relevant improvement (>4 units) in SGRQ was detected in the BDP/FF group only.

**Conclusion:**

BDP/FF extrafine combination provides COPD patients with an equivalent improvement of dyspnoea and a faster bronchodilation in comparison to FP/S.

**Trial registration:**

ClinicalTrials.gov: NCT01245569

## Background

Chronic obstructive pulmonary disease (COPD) is a progressive disease estimated to become the third leading cause of death and the fifth cause of morbidity worldwide by 2020 [1]. It is characterised by progressive airflow limitation which is not fully reversible and is associated with an enhanced pulmonary inflammatory response. Disease severity is determined by the degree of airflow limitation, the frequency of exacerbations, the severity of symptoms and the presence of co-morbidities. Current medical interventions are now increasingly focused on providing relief to symptoms that majorly impact on quality of life, such as dyspnoea and decreased exercise capacity [1]. One particular focus is the effect of treatments in the morning, when COPD symptoms and patients’ ability to perform daily activities appear to be worst [2].

International guidelines recommend that treatment follows a stepwise approach, with the early introduction of bronchodilators for all patients with COPD, and the later addition of an inhaled corticosteroid (ICS) limited to patients with severe airflow limitation and/or frequent exacerbations [1]. Treatment with a long-acting β_2_ agonist (LABA) and ICS can be administered through single combination inhalers. ICS/LABA combinations reduce the frequency of exacerbations and improve lung function to a greater degree than the monocomponents alone [3], and there are beneficial molecular interactions between these drugs that can potentiate their effectiveness [4,5]. However, high ICS doses have been associated with an increased risk of pneumonia [6-8]. Hence, the dose–response relationship of ICS in COPD is currently a matter of debate [1], especially since head-to-head studies comparing different dosages of ICS in COPD are still lacking [9].

The ICS/LABA combination beclomethasone dipropionate 100 μg plus formoterol fumarate 6 μg (BDP/FF) is an extrafine formulation that optimises small particle deposition throughout the bronchial tree, including the small airways; It is licensed for use in asthma, and is being developed as a treatment for COPD [10,11]. FF is a LABA with a rapid onset of action [12], while in contrast the LABA salmeterol has a slow onset of action. Consequently, a potential advantage of FF over salmeterol when used in combination inhalers is that the morning dose can more rapidly improve lung function, and so provide greater benefits for COPD patients who suffer with morning symptoms [13,14].

The aim of the FUTURE trial was to compare the efficacy of two fixed combination therapies that deliver different ICS doses; extrafine BDP/FF versus fluticasone / salmeterol (FP/S) where the daily ICS doses are 400 μg and 1000 μg / day respectively. We measured symptoms and lung function after 12 weeks to compare the efficacy of these treatments. Furthermore, we also investigated the acute bronchodilator effects in the morning to study differences that might be due to the onset of action of the LABAs.

## Methods

### Patients

This study was carried out in 76 outpatient respiratory clinics throughout Europe and included patients aged ≥ 40 years with a diagnosis of moderate to severe COPD. Inclusion criteria were: smoking history ≥ 10 pack years; regular bronchodilator use in the previous 2 months; post-bronchodilator forced expiratory volume measured in the first second (FEV_1_) < 60% of predicted; post-bronchodilator FEV_1_/forced vital capacity (FVC) < 0.7; an increase in FEV_1_ ≥ 5% from baseline following administration of 400 μg of salbutamol; a Baseline Dyspnoea Index (BDI) focal score ≤ 10 at the screening and randomisation, and a history of ≤ one COPD exacerbation treated with antibiotics or systemic corticosteroids in the previous 12 months. Patients were excluded if they had been diagnosed with asthma, other respiratory disorders, or any other clinically relevant condition that could have interfered with the evaluation of results.

The study was performed in accordance with the principles of the Declaration of Helsinki and with the Good Clinical Practice guidelines recommended by the International Conference on Harmonization of Technical Requirements. The protocol was approved by the Institutional Review Board of each centre (a list is shown in Additional file 1), and informed written consent was obtained from each participant prior to study entry.

### Study design

This was a randomised, double-blind, double-dummy, 2-arm parallel group study. After a screening visit, patients entered a 2-week run-in period, where they received inhaled ipratropium bromide (Atrovent® Inhaler CFC-Free 20 μg) as maintentance treatment administered 4 times / day. Patients were then randomised to a 12-week treatment period with either extrafine BDP/FF 100/6 μg in a hydrofluoralkane pressurised metered dose inhaler (pMDI; FOSTER®, Chiesi Farmaceutici, Parma, Italy) or FP/S 500/50 μg, in a dry-powder inhaler (DPI; Seretide®, Accuhaler® GlaxoSmithKline, Middlesex, UK). Randomization was performed according to a pre-determined balanced-block, computer generated, randomisation list stratified by country. BDP/FF was administered as two puffs twice daily (daily dose 400 μg BDP/24 μg FF), while FP/S was administered as one inhalation twice daily (daily dose 1000 μg FP/100 μg S). Clinic visits were performed at monthly intervals. Inhaled rescue salbutamol use was permitted during the whole study period (including run-in), but no other COPD medications were permitted.

### Protocol outcome measures

The two co-primary efficacy variables were Transition Dyspnoea Index (TDI) score at the end of the study (week 12), and Area Under the Curve (AUC_0-30min_) standardized by time of change from pre-dose in FEV_1_ after drug inhalation during the morning of baseline visit. In order to demonstrate the equivalence between BDP/FF and FP/S in terms of TDI, dyspnoea was assessed at baseline with the Baseline Dyspnoea Index (BDI) score and by TDI score at week 12 [15]. Pulmonary function tests (PFTs) were performed, in accordance with ATS/ERS standards [16], at the screening visit before (pre-bronchodilator) and after (post-bronchodilator) salbutamol inhalation and at each clinic visit. At baseline and week 12, PFTs were performed before study drug inhalation (pre-dose) and then 5, 15 and 30 minutes after (post-dose). At week 4 and 8, spirometry was performed at pre-dose only. PFTs were all performed at least 12 hours after the previous evening dose and 6 hours after previous salbutamol use. Each site was provided with the same spirometer FlowScreen® CT that directly transferred PFTs values to the e-CRF.

A diary card was used each morning at home to record COPD symptom scores, the number of inhalations of study medication (run-in medication included) and salbutamol use; the diary card is shown in Additional file 1. Symptoms assessed with the diary card included ability to perform usual daily activities, breathlessness, night waking caused by respiratory symptoms, breathlessness on rising, cough and sputum production; each was assigned a score ranging from 0 (no symptoms) to 3 (worst), giving a maximum total score of 18 / day; this questionnaire has been used previously in a COPD clinical trial [11], but has not been formally validated for this purpose.

Occurrence of COPD exacerbations and adverse events were evaluated by the Investigator at all visits, by diary review and asking the patient. Health status was assessed using the St. George’s Respiratory Questionnaire (SGRQ) [17,18] at baseline and at week 12. All patient-reported outcomes were gathered pre-dose in the morning. The six-minute walking test (6MWT) was carried out following ATS guidelines [19] at pre-dose and post-dose, both at baseline and week 12.

### Statistics

Data are expressed as mean and standard deviation (SD), unless otherwise specified. The study was powered to detect a mean difference between treatments of 0.080 L in the AUC_0-30min_ standardized by time of change from pre-dose in FEV_1_ at baseline visit, assuming a SD of 0.16 L and using a two-sample t-test with two-sided significance level of 0.05 (further details are in Additional file 1). Superiority for AUC_0-30min_ was demonstrated if the two-sided 95% confidence interval (CI) for the adjusted mean difference between the two drugs lied entirely above 0. Equivalence in TDI score was demonstrated if the two-sided 95% CI for the adjusted mean difference lied entirely within the equivalence margins fixed at ± 1, assuming the true mean difference in TDI score between treatments is 0 and the standard deviation is 2.7.

For all parameters, the analysis of covariance, with treatment and country as factors, and baseline value (pre-dose at randomisation visit) as a linear covariate, was applied. All analyses were performed with SAS™ System (SAS Institute Inc, Cary, NC), version 9.2. Statistical significance was set at 0.05 two tailed, and all analyses were performed on the intention-to-treat population (ITT). Imputation of missing data was completed following last observation carried forward method for post-baseline data. According to the current indication for ICS/LABA use in COPD [1], a pre-defined analysis was performed in patients with FEV_1_ < 50% of predicted for all efficacy variables.

## Results

### Patients

**Figure 1:**
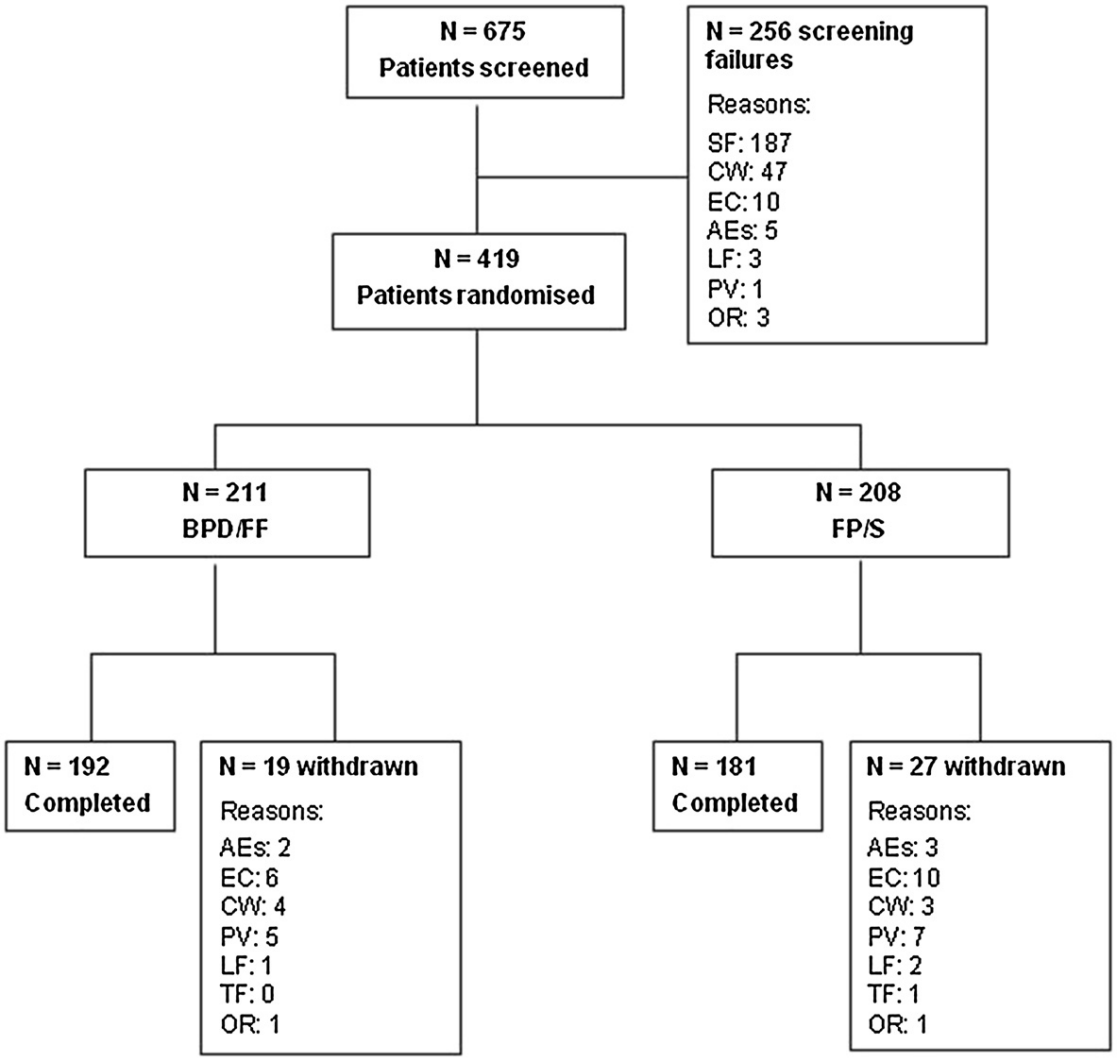
**Patient flow.** SF: screening failure due to ineligibility; CW: consent withdrawn; EC: development of exclusion criteria; AEs: adverse events; LF: lost to follow-up; PV: protocol violations; OR: other reasons; TF: treatment failure. For some AEs (1 in the BDP/FF group and 2 in the FP/S group) the cause of withdrawn was reported as EC, as stated in the Study Termination Form of the e-CRF.

Of the 675 patients screened, 419 were randomized and 373 completed the study (Figure 1). The reasons for screening failure are provided in Additional file 1. The first patient was enrolled in April 2011 and the last completed the trial in March 2012. The most common causes of study discontinuation were development of exclusion criteria (3.8%) and protocol violations (2.9%). The frequency of withdrawal for adverse events was similar in the BDP/FF and FP/S groups (0.9% and 1.4% respectively).

The characteristics of the patients at baseline is summarized in Table 1, (see Additional file 1 for characteristics of the subgroup with FEV_1_ < 50% predicted). Overall, approximately 73% of patients randomised were using ICS at screening. Patient compliance evaluated from diary cards was >98% in both groups.

**Table 1:** Demographics and baseline characteristics

	**BDP/FF**	**FP/S**
	**(N = 211)**	**(N = 207)**
Gender:		
Male (%)	155 (73.5)	143 (69.1)
Female (%)	56 (26.5)	64 (30.9)
Age, *years*	63.8 (8.2)	63.7 (8.6)
BMI, *kg/m*^*2*^	26.16 (5.37)	27.02 (5.60)
Smoking habits:		
Ex-smokers (%)	106 (50.2)	121 (58.5)
Current smokers (%)	105 (49.8)	86 (41.5)
No. of pack years	40.1 (20.4)	41.9 (23.0)
Previous treatments* (%)		
Long-acting anticholinergic	139 (65.9)	140 (63.6)
LABA	51 (24.2)	44 (21.3)
ICS/LABA fixed combination	119 (56.4)	126 (60.9)
ICS	36 (17.1)	28 (13.5)
Pre-dose FEV_1_, *litre*	1.13 (0.40)	1.10 (0.36)
Pre-bronchodilator FEV_1_, *litre*^†^	1.13 (0.33)	1.10 (0.30)
Post- bronchodilator FEV_1_, *litre*^†^	1.31 (0.32)	1.29 (0.33)
Post- bronchodilator FEV_1_/FVC (%)^†^	44.2 (9.7)	44.4 (9.8)
Post- bronchodilator FEV_1_ (% predicted normal)^†^	46.5 (9.6)	46.4 (9.8)
FEV_1_ post-bronchodilator change (%)^†^	17.5 (11.0)	17.8 (11.0)
BDI score	6.1 (1.8)	6.1 (1.7)
SGRQ score	47.0 (16.7)	45.2 (16.5)
pre-dose 6MWT, *metres*	352 (106)	364 (109)
Symptom score	6.27 (3.57)	6.05 (3.72)
Symptom-free days (%)	7.55 (20.14)	7.21 (20.59)
Rescue medication-free days (%)	44.27 (42.10)	39.69 (39.86)
Patients with no exacerbations in the previous year	116 (55.0%)	108 (52.2%)

All values are presented as mean (SD) or absolute number (%).

BDP/FF, beclomethasone dipropionate/formoterol fumarate; FP/S, fluticasone propionate/salmeterol; BMI, body mass index; LABA, long-acting β_2_-agonist; ICS, inhaled corticosteroids; FEV_1_, forced expiratory volume in the first second, FVC, Forced Vital Capacity; BDI, basal dyspnoea index; SGRQ, St. George’s respiratory questionnaire; 6MWT, 6 minutes walking test.

*patients can have more than one treatment. ^†^Evaluated in the screening visit (before and after salbutamol inhalation).

### Primary endpoints

**Figure 2:**
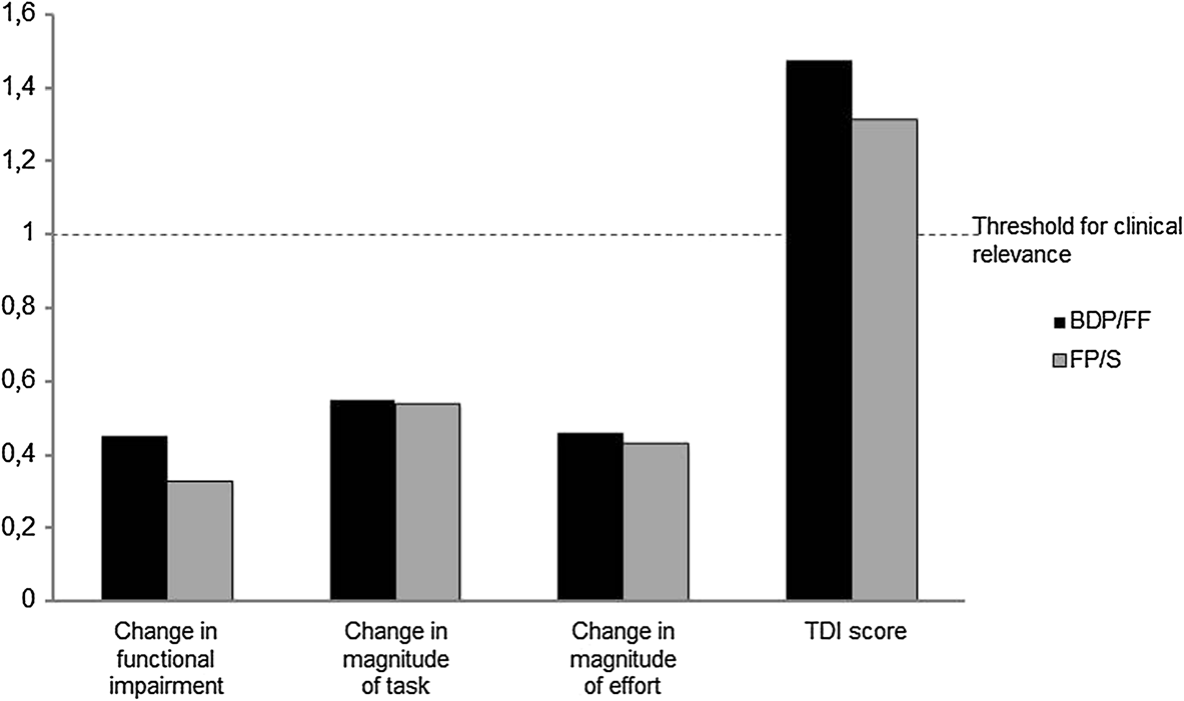
**Transition dyspnoea index (TDI) score at week 12.** BDP/FF, beclomethasone dipropionate/formoterol fumarate; FP/S, fluticasone propionate/salmeterol. There were no differences (p > 0.05) between BDP/FF and FP/S. The threshold for clinical relevance only applies to the TDI total domain score.

At week 12, the TDI score had improved in both groups (Figure 2); the adjusted means (95% CI) were 1.32 (0.87-1.77) for BDP/FF and 1.15 (0.70-1.60) for FP/S; the mean difference between treatments was 0.17 and the 95% CI for the difference (-0.39 to 0.72; p = 0.56) was entirely within the ±1 equivalence margins, with no statistically significant difference between treatments. Ninety-three patients (44.1%) in the BDP/FF group and 89 (43.0%) in the FP/S group had a TDI score ≥ 1 (p = 0.92). The equivalence between BDP/FF and FP/S was also demonstrated in patients with FEV_1_% predicted <50%, where the difference between groups was -0.06 (-0.78 to 0.66; p = 0.87); 43 patients (36.1%) in the BDP/FF group and 50 (41.0%) in the FP/S group (p = 0.51) showed a TDI score ≥ 1.

**Figure 3:**
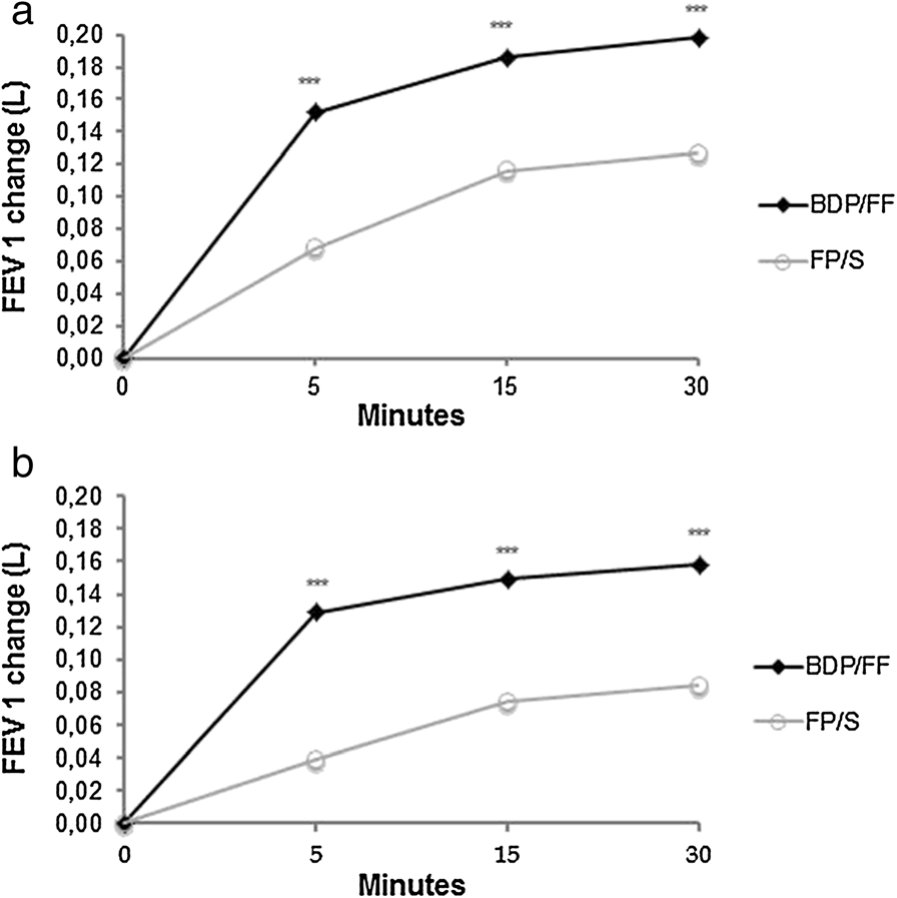
**Change in FEV1. (a)** Change from pre-dose in FEV_1_ (L) measured at baseline; **(b)** Change from pre-dose in FEV_1_ (L) measured at week 12. FEV_1_, forced expiratory volume in the first second; BDP/FF, beclomethasone dipropionate/formoterol fumarate; FP/S, fluticasone propionate/salmeterol. p < 0.001 at each time point compared to baseline pre-dose for both treatments and at each visit. ***p < 0.001 between treatments.

The AUC_0-30min_ adjusted means (95% CI) were 0.18 L (0.16-0.19) and 0.11 L (0.09-0.12) for the BDP/FF and FP/S groups respectively, with a statistically significant difference between groups of 0.07 L (0.05-0.10; p < 0.001). Similarly, in patients with FEV_1_ < 50% of predicted the difference between groups was 0.08 L (0.05-0.10, p < 0.001). Figure 3 shows that FEV_1_ improved significantly at 5, 15 and 30 minutes post-dose in both treatment groups (p < 0.001 at all-time points). The improvements were significantly greater after BDP/FF compared to FP/S group at all timepoints (p < 0.001); the mean (95% CI) differences at 5, 15 and 30 minutes were 0.08 (0.06-0.11); 0.07 (0.04-0.10) and 0.07 (0.04-0.10), respectively.

### Secondary endpoints

There was an increase in the pre-dose morning FEV_1_ at week 12 compared to baseline in both treatments groups, with no difference between treatments; see Table 2 (the analysis of patients with FEV_1_ < 50% predicted is in Additional file 1).

**Table 2:** Comparisons between the groups at week 12: adjusted values from ANCOVA analysis

	**BDP/FF**	**FP/S**	**Between groups p value**
	**(N = 211)**	**(N = 207)**	
Pre-dose morning FEV_1_, *litre*	0.08 (0.04 to 0.11)	0.06 (0.03 to 0.10)	0.58
Pre-dose morning FVC, *litre*	0.06 (-0.00 to 0.13)	0.05 (-0.01 to 0.12)	0.82
SGRQ score	-5.92 (-7.75 to -4.08)	-3.80 (-5.70 to -1.90)	0.08
SGRQ decrease from baseline > 4, patients (%)	95 (45.0)	75 (36.2)	0.16
6MWT^a^, *meters*	31.62 (15.18 to 48.06)	22.23 (6.30 to 38.16)	0.33
6MWT change^a^ > 37 meters, patients (%)	39 (18.8)	34 (16.4)	0.60
Symptom score^b^	-1.21 (-1.55 to -0.87)	-1.00 (-1.35 to -0.65)	0.36
Breathlessness on rising^b^	-0.26 (-0.35 to -0.18)	-0.24 (-0.32 to -0.16)	0.65
Symptom-free days^c^,%	4.60 (1.79 to 7.41)	5.88 (2.99 to 8.76)	0.50
Use of rescue medication^b^, *puffs*	-0.60 (-0.78 to -0.42)	-0.63 (-0.81 to -0.45)	0.80
Rescue medication-free days^c^,%	13.50 (9.39 to 17.61)	13.11 (8.89 to 17.32)	0.89

All values are presented as mean (95% CI) or absolute number (%).

BDP/FF, beclomethasone dipropionate/formoterol fumarate; FP/S, fluticasone propionate/salmeterol; FEV_1_, forced expiratory volume in the first second; FVC, Forced Vital Capacity; SGRQ, St. George’s respiratory questionnaire 6MWT, 6 minutes walking test.

^a^change from pre-dose at randomization visit to pre-dose at the end of treatment.

^b^mean of the last two weeks (week 11–12).

^c^overall results were considered (entire treatment period).

FEV_1_ post-dose at week 12 improved significantly in both treatment groups (p < 0.001 at all time-points; Figure 3b), with a significantly greater effect observed with BDP/FF at all-time points (p < 0.001 at each time-point). The mean differences (95% CI) between groups at 5, 15 and 30 minutes were 0.09 (0.07-0.11); 0.08 (0.05-0.10) and 0.07 (0.05-0.10), respectively. Similar changes were seen in patients with FEV_1_ predicted <50%; see Additional file 1 for figure and statistical analysis.

COPD total symptom score decreased significantly (p < 0.001) from baseline in both treatment groups at all intermediate visits (data not shown) and at the end of the study (see Additional file 1), with no difference between treatments (see Table 2). The number of symptom-free days increased significantly in both groups (p < 0.001), with no difference between groups. There were similar findings in patients with FEV_1_ predicted <50% (see Additional file 1).

**Figure 4:**
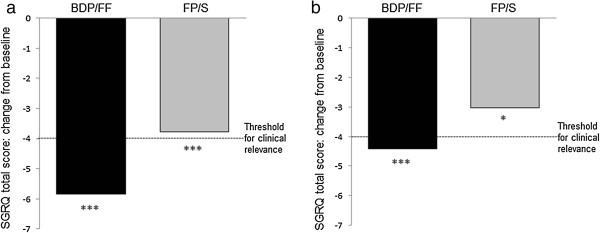
**Change in SGRQ. (a)** Change from baseline in SGRQ total score; **(b)** Change from baseline in SGRQ total score in the ITT population with FEV_1_ < 50% of predicted normal value. SGRQ, St. George Respiratory Questionnaire; BDP/FF, beclomethasone dipropionate/formoterol fumarate; FP/S, fluticasone propionate/salmeterol. p > 0.05 (not significant) for differences between BDP/FF and FP/S both in the general and in the FEV_1_ < 50% of predicted normal value population. P values shown refer to change from baseline: ***p < 0.001, and *p = 0.01.

The SGRQ total score, and the single domain scores, decreased significantly from baseline to week 12 (p < 0.001) (see Figure 4 and Additional file 1), with no significant difference between groups (Table 2). The pre-dose distance walked in 6 minutes increased significantly in both treatment groups with respect to baseline in both groups (see Additional file 1). The difference between groups in the change from baseline to week 12 was 9.39 metres (95% CI: -9.63 to 28.41), and was not statistically significant (Table 2). There were similar findings in patients with FEV_1_ predicted <50%; see Additional file 1.

Six (2.8%) patients in the BDP/FF treatment group and 4 (1.9%) in the FP/S group reported exacerbations, with no difference between groups. The majority of exacerbations occurred in patients with FEV_1_% < 50% of predicted; 5 (4.2%) patients in the BDP/FF group and 3 (2.5%) in the FP/S group.

### Tolerability

Treatment-emergent serious adverse events (SAEs) were significantly lower (p = 0.024) in the BDP/FF group (4 patients, 1.9%) than in the FP/S group (13 patients, 6.3%). Pneumonia was reported in 3 patients (1.4%) treated with FP/S and none treated with BDP/FF. Worsening of COPD was reported in 2 (1.0%) patients treated with FP/S and none treated with BDP/FF. Three patients (1.4%) treated with BDP/FF and 5 treated with FP/S (2.4%) discontinued the study due to adverse events (AEs). There were no differences between groups in terms of vital signs and ECG. Further details of adverse events are in Additional file 1.

## Discussion

This is the first head-to-head study comparing extrafine BDP/FF with one of the most commonly used drugs for COPD patients (FP/S), evaluating different ICS dosages in fixed combination therapies. The TDI findings demonstrate that the lower ICS dose in the extrafine BDP/FF combination compared to FP/S provides equivalent symptom control, while the lung function findings for AUC_0-30min_ confirmed a faster onset of action of the formoterol component compared to salmeterol.

The co-primary endpoint measurement of TDI assessed changes in breathlessness that may have been due either to differences in the ICS or LABA in the combination inhalers. We have demonstrated that the BDP/FF combination with a lower ICS dose compared to the FP/S combination was associated with similar improvements in symptom control. This may be due to the effects of the LABAs within these combination inhalers [3,20] as well as to better distribution of extrafine particles of ICS. There are differences between these LABAs, as we have demonstrated for the AUC_0-30min_, and others have also reported in COPD studies of combination inhalers [13,14]. It is difficult in head to head studies of combination inhalers to be sure which effect is due to the LABA or ICS. Nevertheless, the similar results for both combinations suggests that the ICS dose used does not influence symptom control.

A major goal of COPD pharmacotherapy is to relieve subjective symptoms [1]. The equivalence of the TDI score demonstrates that extrafine BDP/FF impacts COPD symptoms to the same degree as FP/S, despite using a lower ICS dose. A 1 unit change in TDI is recognised as a clinically meaningful difference, so we chose this magnitude of change for the equivalence limits [21,22]. We also analysed the number of patients who improved by >1, and again did not find any difference between the treatments.

Early morning symptoms are very common in COPD patients [2,23]. Rapid bronchodilation improves physical exercise tolerance upon wakening, which is one of the most common COPD-associated symptoms [23]. The faster onset of action of formoterol compared to salmeterol has been clearly demonstrated in asthma [24], but results in COPD patients are less consistent, as previous studies comparing these drugs in COPD patients have often involved relatively small study populations [25,26], or provided uncertain results [27,28]. However, larger COPD studies have shown that the fixed combination of budesonide/formoterol has a faster onset of action in the morning compared to FP/S [13,14]. We now also demonstrate this finding for extrafine BDP/FF, and show that this is present after the first dose and maintained after 12 weeks treatment. The benefit achieved after the first dose may be important in providing patients with reassurance regarding drug effectiveness, which will likely improve medication compliance [29]. The maintained presence of a faster onset of action in the morning observed up to 12 weeks may benefit COPD patients who chronically suffer with early morning symptoms.

The secondary efficacy endpoint measurements, which included changes in FEV_1_ pre-dose, SGRQ and 6MWT, also showed no difference between treatments. Both treatments improved SGRQ scores and 6MWT compared to baseline, although only the mean SGRQ change from baseline for BDP/FF reached the clinical meaningful threshold (> 4 units). A large randomized controlled COPD study [11] reported similar efficacy of the BDP/FF extrafine combination to the higher ICS dose in the budesonide/formoterol combination on the 6MWT over 48 weeks. The important role of long acting bronchodilators in reducing hyperinflation and thus improving exercise tolerance is well established, and these 6MWT results are likely to reflect similarities in the degree of bronchodilation and benefit on hyperinflation achieved by the LABAs [30].

The effectiveness of the lower ICS dose in BDP/FF may be due to the extra-fine formulation that allows homogeneous distribution of the two drugs throughout the bronchial tree [31] entailing a greater efficacy per microgram of ICS, in agreement with previous studies on asthma [32-34]. The extra-fine BDP/FF formulation allows the treatment of both large and small airways; the latter are particularly relevant in COPD pathophysiology [35]. Peripheral airway obstruction causes progressive “air trapping” during exercise and consequent limitation of exercise capacity in COPD patients [1]. The effect of BDP/FF extrafine combination on small airways in COPD has been demonstrated in terms of reduction of air trapping measured as reduction in residual volume [36] and increase in FVC [11].

The use of a combination with a lower ICS dose can be of particular relevance in COPD patients since side effects of ICS are dose-dependent [7] and are linked to an increased risk of pneumonia, as highlighted in the TORCH study [20]. Moreover, the approved dosage of FP/S in COPD patients is 500 μg/50 μg twice daily in the EU [37], while in the US only a lower dose (250 μg/50 μg twice daily) was approved on the basis of clinical trial results [38-40], and importantly because an efficacy advantage of the higher strength had not been demonstrated [41].

One limitation of this study may be the lack of a third arm as a control group (e.g. LABA alone). However, the superiority of both extrafine BDP/FF and FP/S over placebo and the monocomponents have been previously demonstrated [11,20].

The COPD patients enrolled were required to have FEV_1_ < 60% of predicted, as ICS/LABA combination therapies are most commonly used in patients with more severe airflow obstruction, and the license for FP/S matches this pulmonary function criteria. ICS/LABA combinations are licensed for use in patients with greater than or equal to one exacerbation per year. We excluded patients with greater than one exacerbation per year, in order to avoid a high drop out rate due to exacerbations during the trial period. Patients were required to demonstrate an increase in FEV_1_ ≥ 5% after salbutamol at screening; this ensured that the patients had some degree of therapeutic response to beta-agonist treatment. We considered this to be a useful inclusion criteria when comparing long acting beta agonists [13,28]. It should be noted that 73.5% of the patients included in this study were using ICS before entry into the study; the population enrolled therefore was highly representative of patients who use ICS in real life.

## Conclusions

The present study shows that, despite its lower ICS dose, extrafine BDP/FF (200/12 μg twice daily) is equivalent in improving dyspnoea and has a faster onset of action with respect to FP/S (500/50 μg twice daily) in patients with COPD. The benefits for patients treated with extrafine BDP/FF compared to FP/S may be twofold: first, the lower concern related to ICS dosage and second, a faster onset of bronchodilation, which can reduce morning symptoms.

## Competing interests

D. Singh has received lecture fees, research grants, consultancy fees and support for conference attendance from various pharmaceutical companies including AstraZeneca, GlaxoSmithKline, Chiesi, Boehringer Ingleheim, Roche, Novartis, Cipla, Almirall and Merck. G. Nicolini, E. Bindi and D.Guastalla are employees of Chiesi Farmaceutici S.p.A. M. Corradi has provided consultancy services to Chiesi Farmaceutici. J. Kampschulte, W. Pierzchala and M.Szilasi have nothing to disclose. A. Sayiner has received honoraria for consulting and presenting from AstraZeneca, Boehringer Ingelheim, GlaxoSmithKline, Novartis and Pfizer. C. Terzano has received honoraria for consulting and presenting from Chiesi Farmaceutici, Menarini, AstraZeneca, Boehringer Ingelheim, GlaxoSmithKline, Pfizer, Novartis, Artsana and Mefar. J. Vestbo has received honoraria for consulting and presenting from Chiesi Farmaceutici, AstraZeneca, Bioxydyn, Boehringer Ingelheim, GlaxoSmithKline, Novartis, Syntaxin and Takeda.

## Authors’ contributions

DS, JV and GN were involved in study conception and design, data interpretation, manuscript writing and approval. EB, MC and DG were involved in study conception and design, data interpretation and manuscript approval. JK, WP, AS, MS and CT were involved in study design, data acquisition, manuscript revision and manuscript approval. All authors read approved the final manuscript.

## Pre-publication history

The pre-publication history for this paper can be accessed here:

http://www.biomedcentral.com/1471-2466/14/43/prepub

## Supplementary Material

Additional file 1 **Contains supplementary methods and results including: List of institutional review boards; Reasons for screen failure; Demographics of patients with FEV1<50% of predicted; Change from baseline at week 12 in efficacy parameters; Comparisons between treatments in patients with FEV**_**1**_**<50% of predicted; Symptom diary card; Change in FEV1 in patients with FEV1 <50% predicted.** Click here for file
