# Supplementary material for: Extrafine Beclomethasone/formoterol compared to Fluticasone/salmeterol Combination Therapy in COPD
Source: BMC Pulm Med. 2014 Mar 12;14:43. doi: 10.1186/1471-2466-14-43 (PMC4008134; doi:10.1186/1471-2466-14-43)
Supplement: Additional file 1 — Contains supplementary methods and results including: List of institutional review boards; Reasons for screen failure; Demographics of patients with FEV1<50% of predicted; Change from baseline at week 12 in efficacy parameters; Comparisons between treatments in patients with FEV 1 <50% of predicted; Symptom diary card; Change in FEV1 in patients with FEV1 <50% predicted. [file 1471-2466-14-43-S1.doc]

**Additional file 1**

**METHODS**

**Patients**

A list of the IRBs is shown below:

| **Country** | **Sites** | **IECs/IRBs** |
| --- | --- | --- |
| Denmark | All Sites | The Biomedical Research Ethics Committees  for the Capital Region Regionsgården Group Secretariat  Kongens Vænge 2, 3400 Hillerød |
| France | All Sites | Comité de Protection des Personnes  CPP Nord Ouest IV et Pharmacology – Faculte de Medecine  1 Place de Verdun, 59045 Lille Cedex |
| Germany | All Sites | Landesamt für Gesundheit und Soziales Berlin  Geschäftsstelle der Ethik-Kommission des Landes Berlin  Sächsische Straße 28, 10707 Berlin |
| Hungary | All Sites | Medical Research Council, Ethics Committee for Clinical Pharmacology  Arany J. u. 6-8, 1051 Budapest |
| Italy | All Sites | Comitato Etico dell’ Azienda Policlinico Umberto I di Roma  Viale del Policlinico, 155 – 00161 Rome |
| Poland | All Sites | ŚLĄSKA IZBA LEKARSKAKOMISJA BIOETYCZNA  Bioethics Committee of Slaska Izba Lekarska in Katowice  ul. Grażyńskiegi, 40-126 Katowice |
| Slovakia | Site 0701 | Eticka Komisia Kosickeho Samospravneho Kraja  Námestie Maratónu Mieru 1, 04266 Košice |
|  | Site 0702 | Ethics Committee of Trnava Self Governing Region  Starohájska 10, 91701 Trnava |
|  | Site 0703 | Ethics Committee of Trenčín Self Governing Region  K dolnej stanici 7282/20A,91101 Trenčín |
|  | Site 0704 | Etická Komisia Nitrianskeho Samosprávneho Kraja.  Stefániková trieda c . 69. 949 60 Nitra |
|  | Site 0705, 0706, 0707, 0708 | Eticka Komisia Presovského Samosprávneho Kraja  Namestie Mieru 2, 08001 Presov |
| Spain | All Sites | Coordinator Ethics Committee (CEC)  CEIC Coporació Sanitaria Parc Taulí  Parc Taulí, 1. 08208 Sabadell. Barcelona |
| Turkey | All Sites | Republic of Turkey – Ege University Faculty of Medicine  Clinic Research Ethics Committee  Ege Universitesi Tip Fakultesi Dekanligi 2.  Kat Erzene Ankara Cad. 35100 Bornova / IZMIR |
| United Kingdom | All Sites | North West Centre of Research Ethics Committees  Barlow House, 4 Minshull Street, Manchester M1 3DZ |

**Measurements**

Heart rate, systolic and diastolic blood pressure in sitting position were measured at each visit. 12-lead ECG was performed at screening visit and at the end of treatment.

**Statistics**

The study aimed to prove both equivalence of drugs in terms of TDI at week 12, and superiority of BDP/FF versus FP/S in terms of AUC_0-30min_ standardized by time of change from pre-dose in FEV1 at baseline visit. Because both endpoints had to be met for the trial to be successful, no formal adjustment of the significance level was necessary, but the power requested for each single test (equivalence and superiority) was set in order to assure an overall 85% power for the study.

**Results**

**Tolerability**

One patient affected by tachycardia in the group treated with BDP/FF, and one by cough in the FP/S group were considered treatment-related non-serious events, while all other AEs leading to study discontinuation were considered as non-treatment related. One patient in the BDP/FF group died due to acute myocardial infarction, which was considered as not related to study medication.

**Table 1A .** Demographics and baseline characteristics of patients with FEV1<50% of predicted.

|  | **BDP/FF**  **(N = 119)** | **FP/S**  **(N = 122)** |
| --- | --- | --- |
| Gender:  Male (%)  Female (%) | 90 (75.6)  29 (24.4) | 89 (73.0)  33 (27.0) |
| Age, *years* | 63.3 (8.6) | 63.1 (8.3) |
| BMI, *kg/m^2^* | 25.6 (5.5) | 26.4 (5.2) |
| Smoking habits:  Ex-smokers (%)  Current smokers (%)  No. of pack/year | 59 (49.6)  60 (50.4)  41.2 (19.4) | 69 (56.6)  53 (43.4)  42.5 (22.8) |
| Previous treatments* (%) |  |  |
| long-acting anticholinergic | 87 (73.1) | 93 (76.2) |
| LABA | 29 (24.4) | 23 (18.8) |
| ICS/LABA fixed combination | 68 (73.9) | 77 (63.1) |
| ICS | 22 (18.5) | 15 (12.3) |
| Pre-dose FEV_1_, *litre* | 0.97 (0.3) | 0.98 (0.3) |
| Pre-bronchodilator FEV_1_, *litre*^†^ | 0.97 (0.3) | 0.98 (0.3) |
| Post-bronchodilator FEV_1_, *litre*^†^ | 1.14 (0.3) | 1.15 (0.3) |
| Post-bronchodilator FEV_1/_FVC (%)^†^ | 40.92 (9.1) | 40.42 (8.3) |
| Post-bronchodilator FEV_1_ (% predicted normal) ^†^ | 39.90 (7.3) | 40.12 (7.4) |
| BDI score | 5.9 (7.1) | 6.1 (1.6) |
| SGRQ score | 48.77 (16.0) | 43.11 (17.1) |
| pre-dose 6MWT, *metres* | 349.7 (110.6) | 344.6 (100.5) |
| Symptom score | 6.67 (3.40) | 6.58 (3.59) |
| Symptom-free days (%) | 5.93 (17.6) | 3.73 (13.6) |
| Rescue medication-free days (%) | 40.72 (41.2) | 34.93 (39.1) |

All values are presented as mean (SD) or absolute number (%).

BDP/FF, beclomethasone dipropionate/formoterol fumarate; FP/S, fluticasone propionate/salmeterol; BMI, body mass index; LABA, long-acting β_2_-agonist; ICS, inhaled corticosteroids; FEV_1_, forced expiratory volume in the first second; FVC, Forced Vital Capacity; BDI, basal dyspnoea index; SGRQ, St. George’s respiratory questionnaire; 6MWT, 6 minutes walking test.

* Patients can have more than one treatment. ^†^ Evaluated at the screening visit.

**Table 2A** Change from baseline at week 12 in efficacy parameters.

|  | **BDP/FF**  **(N = 211)** | **p value** | **FP/S**  **(N = 207)** | **p value** |
| --- | --- | --- | --- | --- |
| Pre-dose morning FEV_1_, *litre* | 0.07 (0.23) | <0.001 | 0.06 (0.21) | <0.001 |
| Pre-dose morning FVC, *litre* | 0.04 (0.47) | 0.291 | 0.05 (0.45) | 0.153 |
| SGRQ score | -5.8 (12.0) | <0.001 | -3.8 (12.1) | <0.001 |
| SGRQ decrease from baseline > 4, patients (%) | 95 (45.0) | - | 75 (36.2) | - |
| 6MWT^a^, *meters* | 27.5 (91.1) | 0.001 | 15.4 (61.3) | 0.008 |
| 6MWT^a^ change > 37 meters, patients (%) | 39 (18.8) | - | 34 (16.4) | - |
| Symptom score | -1.5 (2.3) | <0.001 | -1.2 (2.6) | <0.001 |
| Symptom-free days (%)^b^ | 4.3 (18.1) | <0.001 | 5.7 (19.9) | <0.001 |
| Use of rescue medication, *puffs* | -0.7 (1.5) | <0.001 | -0.7 (1.4) | <0.001 |
| Rescue medication-free days (%)^b^ | 13.5 (30.1) | <0.001 | 14.8 (30.0) | <0.001 |

All values are presented as mean (SD) or absolute number (%).

BDP/FF, beclomethasone dipropionate/formoterol fumarate; FP/S, fluticasone propionate/salmeterol; FEV_1_, forced expiratory volume in the first second; FVC, Forced Vital Capacity; SGRQ, St. George’s respiratory questionnaire 6MWT, 6 minutes walking test.

^a^ change from pre-dose at randomization visit to pre-dose at the end of treatment

^b^ overall results were considered (entire treatment period).

**Table 3A.** Change from baseline at week 12 in efficacy parameters in patients with FEV1<50% of predicted.

|  | **BDP/FF**  **(N = 119)** | **p value** | **FP/S**  **(N = 122)** | **p value** |
| --- | --- | --- | --- | --- |
| Pre-dose morning FEV_1_, *litre* | 0.03 (0.20) | 0.134 | 0.05 (0.20) | 0.006 |
| Pre-dose morning FVC, *litre* | -0.01 (0.42) | 0.877 | 0.03 (0.43) | 0.459 |
| SGRQ score | -4.4 (11.8) | <0.001 | -3.0 (11.7) | 0.010 |
| SGRQ decrease from baseline > 4, patients (%) | 19 (7.8) | - | 26 (10.7) | - |
| 6MWT^a^, *meters* | 16.2 (68.7) | 0.067 | 24.7 (52.0) | <0.001 |
| 6MWT^a^ change > 37 meters, patients (%) | 18 (14.8) | - | 24 (19.7) | - |
| Symptom score | -1.3 (2.3) | <0.001 | -1.1 (2.5) | <0.001 |
| Symptom-free days (%)^b^ | 3.3 (19.2) | 0.066 | 4.5 (16.9) | 0.004 |
| Use of rescue medication, *puffs* | -0.7 (1.6) | <0.001 | -0.7 (1.6) | <0.001 |
| Rescue medication-free days (%)^b^ | 10.7 (28.2) | <0.001 | 13.5 (30.0) | <0.001 |

All values are presented as absolute number (%) or mean (SD).

BDP/FF, beclomethasone dipropionate/formoterol fumarate; FP/S, fluticasone propionate/salmeterol; FEV_1_, forced expiratory volume in the first second; FVC, Forced Vital Capacity; SGRQ, St. George’s respiratory questionnaire 6MWT, 6 minutes walking test.

^a^ change from pre-dose at randomization visit to pre-dose at the end of treatment

^b^ overall results were considered (entire treatment period).

In patients with FEV_1_< 50% of predicted, post-dose FEV_1_ at week 12 improved significantly in both treatment groups (p<0.001 at all timepoints), with a significantly greater effect observed with BDP/FF at any time point (p<0.001 at week 4, p = 0.009 at week 8 and p = 0.016 at week 12).

**Table 4A.** Comparisons between the groups at week 12 in patients with FEV_1_<50% of predicted: adjusted values from ANCOVA analysis.

|  | **BDP/FF**  **(N = 119)** | **FP/S**  **(N = 122)** | **Between groups**  **p value** |
| --- | --- | --- | --- |
| Pre-dose morning FEV_1_, *litre* | 0.04 (0.00 to 0.07) | 0.06 (0.03 to 0.10) | 0.288 |
| Pre-dose morning FVC, *litre* | 0.003 (-0.07 to 0.08) | 0.05 (-0.03 to 0.13) | 0.349 |
| SGRQ score | -4.61 (-6.95 to -2.27) | -3.54 (-5.94 to -1.14) | 0.490 |
| SGRQ decrease from baseline > 4, patients (%) | 19 (7.8) | 26 (10.7) | 0.237 |
| 6MWT^a^, *meters* | 25.30 (5.92 to 44.67) | 31.83 (13.12 to 50.54) | 0.537 |
| 6MWT change^a^ > 37 meters, patients (%) | 18 (14.8) | 24 (19.7) | 0.314 |
| Symptom score^b^ | -1.05 (-1.52 to -0.58) | -0.79 (-1.27 to -0.32) | 0.428 |
| Symptom-free days^c^ | 4.10 (0.64 to 7.56) | 4.35 (0.79 to 7.90) | 0.916 |
| Use of rescue medication^b^, *puffs* | -0.58 (-0.84 to -0.31) | -0.72 (-0.98 to -0.45) | 0.439 |
| Rescue medication-free days^c^ | 11.80 (6.48 to 17.13) | 13.04 (7.54 to 18.55) | 0.733 |

All values are presented as mean (95% CI) absolute number (%).

BDP/FF, beclomethasone dipropionate/formoterol fumarate; FP/S, fluticasone propionate/salmeterol; FEV_1_, forced expiratory volume in the first second; FVC, Forced Vital Capacity; SGRQ, St. George’s respiratory questionnaire 6MWT, 6 minutes walking test.

^a^ change from pre-dose at randomization visit to pre-dose at the end of treatment

^b^ mean of the last two weeks (week 11-12)

^c^ overall results were considered (entire treatment period).

**Diary card to assess COPD Symptoms**

| **COPD SYMPTOMS**  Please complete in the morning and cross the appropriate score referred to the statemtns below (it can be 0 – 1 – 2 – 3) during the previous 24 hr, according to the following scale:  **0 = no symptoms 3 = the worst possible** | | | | | | | | | | | | | | | | | | | | | | | | | | | | |
| --- | --- | --- | --- | --- | --- | --- | --- | --- | --- | --- | --- | --- | --- | --- | --- | --- | --- | --- | --- | --- | --- | --- | --- | --- | --- | --- | --- | --- |
| **Day No.** | **Day 1** | | | | **Day 2** | | | | **Day 3** | | | | **Day 4** | | | | **Day 5** | | | | **Day 6** | | | | **Day 7** | | | |
| Ability to perform the usual daily activities | 0 | 1 | 2 | 3 | 0 | 1 | 2 | 3 | 0 | 1 | 2 | 3 | 0 | 1 | 2 | 3 | 0 | 1 | 2 | 3 | 0 | 1 | 2 | 3 | 0 | 1 | 2 | 3 |
| Breathlessness | 0 | 1 | 2 | 3 | 0 | 1 | 2 | 3 | 0 | 1 | 2 | 3 | 0 | 1 | 2 | 3 | 0 | 1 | 2 | 3 | 0 | 1 | 2 | 3 | 0 | 1 | 2 | 3 |
| Waking at night due to respiratory symptoms | 0 | 1 | 2 | 3 | 0 | 1 | 2 | 3 | 0 | 1 | 2 | 3 | 0 | 1 | 2 | 3 | 0 | 1 | 2 | 3 | 0 | 1 | 2 | 3 | 0 | 1 | 2 | 3 |
| Breathlessness on rising | 0 | 1 | 2 | 3 | 0 | 1 | 2 | 3 | 0 | 1 | 2 | 3 | 0 | 1 | 2 | 3 | 0 | 1 | 2 | 3 | 0 | 1 | 2 | 3 | 0 | 1 | 2 | 3 |
| Cough | 0 | 1 | 2 | 3 | 0 | 1 | 2 | 3 | 0 | 1 | 2 | 3 | 0 | 1 | 2 | 3 | 0 | 1 | 2 | 3 | 0 | 1 | 2 | 3 | 0 | 1 | 2 | 3 |
| Sputum production | 0 | 1 | 2 | 3 | 0 | 1 | 2 | 3 | 0 | 1 | 2 | 3 | 0 | 1 | 2 | 3 | 0 | 1 | 2 | 3 | 0 | 1 | 2 | 3 | 0 | 1 | 2 | 3 |

| **“RESCUE” MEDICATION – you should use the “rescue” drug only if needed**  Please complete in the morning and enter the number of puffs of the “rescue” medication you have taken during the previous 24h. If you did not take any puffs, please enter 0 (zero). | | | | | | | |
| --- | --- | --- | --- | --- | --- | --- | --- |
| **Day No.** | **Day 1** | **Day 2** | **Day 3** | **Day 4** | **Day 5** | **Day 6** | **Day 7** |
| Salbutamol pMDI |  |  |  |  |  |  |  |

**On-line Figure 1A legend**


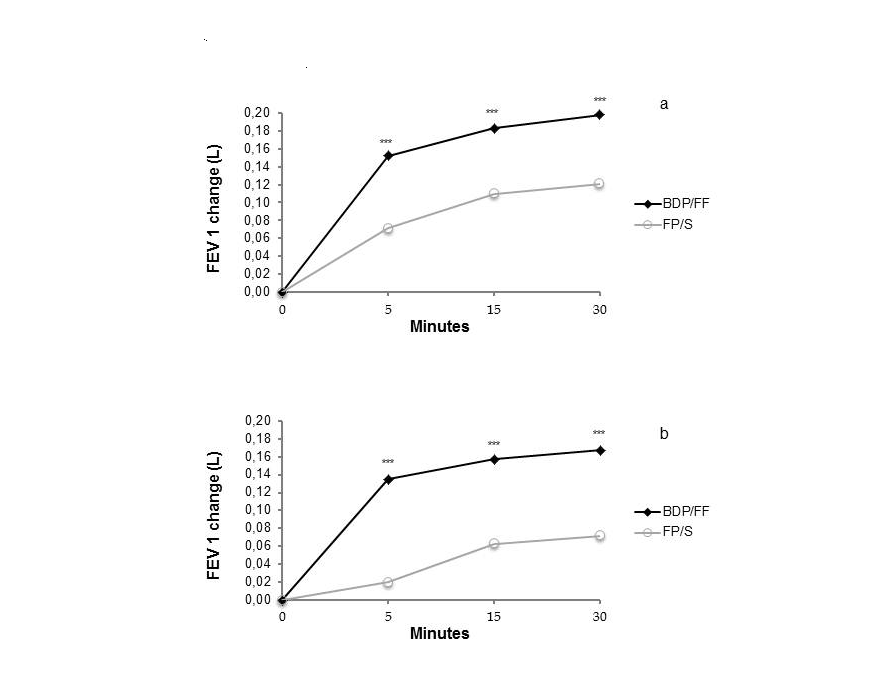


Both figures are related to the ITT population with FEV_1_ < 50% of predicted normal value. (a) Change from pre-dose in FEV_1_ (L) measured at baseline; (b) Change from pre-dose in FEV_1_ (L) measured at week 12 FEV_1_

BDP/FF = beclomethasone dipropionate/formoterol fumarate; FP/S = fluticasone propionate/salmeterol. p < 0.001 at each time point compared to baseline pre-dose for BDP/FF at each visit, p < 0.001 for FP/S at each time point compared to baseline pre-dose at baseline (a) and p=0.004 at week 12 (b). *** p < 0.001 between treatments.
